# Paternal nutritional programming of lipid metabolism is propagated through sperm and seminal plasma

**DOI:** 10.1007/s11306-022-01869-9

**Published:** 2022-02-10

**Authors:** Samuel Furse, Adam J. Watkins, Huw E. L. Williams, Stuart G. Snowden, Davide Chiarugi, Albert Koulman

**Affiliations:** 1 Core Metabolomics and Lipidomics Laboratory, Wellcome Trust-MRL Institute of Metabolic Science, University of Cambridge, Addenbrooke’s Treatment Centre, Keith Day Road, Cambridge, CB2 0QQ UK; 2 Metabolic Disease Unit, Wellcome Trust-MRL Institute of Metabolic Science, University of Cambridge, Addenbrooke’s Treatment Centre, Keith Day Road, Cambridge, CB2 0QQ UK; 3 Biological Chemistry Group, Jodrell Laboratory, Royal Botanic Gardens Kew, Richmond, UK; 4 Division of Child Health, Obstetrics and Gynaecology, Faculty of Medicine, University of Nottingham, Nottingham, NG7 2UH UK; 5 Biodiscovery Institute, University of Nottingham, University Park, Nottingham, NG7 2RD UK; 6 Department of Biological Sciences, Royal Holloway College, University of London, Egham, TW20 0EX Surrey UK; 7 Bioinformatics and Biostatistics Core, Wellcome Trust-MRL Institute of Metabolic Science, University of Cambridge, Addenbrooke’s Treatment Centre, Keith Day Road, Cambridge, CB2 0QQ UK

**Keywords:** Nutritional programming, Lipid metabolism, Lipid traffic analysis

## Abstract

**Background:**

The paternal diet affects lipid metabolism in offspring for at least two generations through nutritional programming. However, we do not know how this is propagated to the offspring.

**Objectives:**

We tested the hypothesis that the changes in lipid metabolism that are driven by paternal diet are propagated through spermatozoa and not seminal plasma.

**Methods:**

We applied an updated, purpose-built computational network analysis tool to characterise control of lipid metabolism systemically (Lipid Traffic Analysis v2.3) on a known mouse model of paternal nutritional programming.

**Results:**

The analysis showed that the two possible routes for programming effects, the sperm (genes) and seminal plasma (influence on the uterine environment), both have a distinct effect on the offspring’s lipid metabolism. Further, the programming effects in offspring suggest that changes in lipid distribution are more important than alterations in lipid biosynthesis.

**Conclusions:**

These results show how the uterine environment and genes both affect lipid metabolism in offspring, enhancing our understanding of the link between parental diet and metabolism in offspring.

**Supplementary Information:**

The online version contains supplementary material available at 10.1007/s11306-022-01869-9.

## Background

There is growing recognition that paternal nutritional programming is an important factor in the metabolism and health of offspring(Hur et al., 2017; Li et al., 2016; McPherson et al., 2016; Morgan et al., 2020; Watkins & Sinclair, 2014; Watkins et al., 2017, 2018). This research has contributed to the understanding that obesity in both men and women has long-term consequences for their offspring through nutritional programming (Jazwiec & Sloboda, 2019; Tarry-Adkins & Ozanne, 2017; Watkins & Sinclair, 2014; Watkins et al., 2018). It has sparked interest in the mechanisms that lead to nutritional programming. One important avenue is the evidence that lipid metabolism is clearly associated with nutritional programming (Furse et al., 2019, 2021b) and changes in lipid metabolism are associated with an increased risk of cardio-metabolic disease (CMD) (Cropley et al., 2016; Fernandez-Twinn et al., 2019; Ng et al., 2010; Perng et al., 2019; Wei et al., 2014). Lipid metabolism is both shaped by nutritional programming and associated with metabolic ill-health, which suggests shared underlying mechanisms.

However, there are myriad possible mechanisms that can play a role in paternal nutritional programming. First, the nutrition of the parent(s) affects their own body composition and organ function. This includes general effects like obesity of course but also subtler ones such as a high carbohydrate diet increasing the rate of *de novo* lipogenesis. Second, the molecular changes that occur in the offspring as a result of that parental nutrition, such as their volume of adipose (Lecoutre & Breton, 2014; Lukaszewski et al., 2013) and hyperglycaemia (Fernandez-Twinn et al., 2019). Such effects have also been investigated by network analysis of lipid metabolism (Furse et al., 2021b). Third, exposure to poor nutrition or over nutrition can result in epigenetic changes that can also be propagated. However, it remains unclear through which route(s) nutritional programming is propagated, especially from the father to the offspring. Propagation of nutritional programming from fathers to offspring is likely to be simpler than that from mothers to offspring; propagation of paternal programming can only happen through the spermatozoa or seminal plasma. Current evidence suggests that these two have separate effects(Li et al., 2016; McPherson et al., 2016; Morgan et al., 2020; Watkins & Sinclair, 2014; Watkins et al., 2017, 2018; Wei et al., 2014), however a relationship with lipid metabolism has not been reported for either of them individually.

**Fig. 1:**
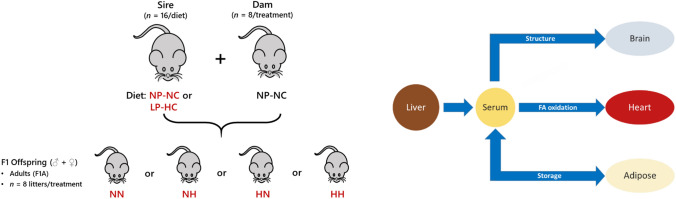
The mouse model and tissues used in the present study. Panel **A**, Schematic representation of the mouse model showing the generation of offspring across two generations. Panel **B**, the network that describes the lipid traffic associated with *de novo* lipogenesis from the liver to termini (CNS, heart and adipose) *via* the serum. The termini represent traffic flow for structural purposes (CNS), fatty acid oxidation (heart) and storage (adipose). This metabolic relationship between tissues was used as the structure of the network for all analyses in the present study. NP-NC refers to a diet of normal protein-normal carbohydrate (NN in the text) whereas LP-HC refers to a low protein-high carbohydrate diet (HH in the text). Adipose was only available for F1A groups, whole brain samples used for F1N groups, with separate right brain and cerebellum for F1A. Figure adapted from reference(Furse et al., 2021b)

In this study we elected to investigate the propagation of paternal nutritional programming effects through spermatozoa and seminal plasma. The hypothesis was that changes in lipid metabolism were driven by paternal diet are propagated through spermatozoa. We tested this using a mouse model of high carbohydrate intake that comprised four feeding groups. The four groups were bred from sperm and seminal plasma from males either fed the normal (N) or high carbohydrate (H) diet (Watkins & Sinclair, 2014; Watkins et al., 2017, 2018). The four groups were represented by two-letter codes, NN, NH, HH and HN, that denoted the origin of the spermatozoon and seminal plasma, respectively (Fig. 1A). Thus group NH was bred from sperm from males fed a normal diet and seminal plasma from a male fed a high carbohydrate diet (H). The groups with both sperm and seminal plasma from sires fed the same diet (NN and HH) were regarded as controls.

We collected the tissues that were most relevant for lipid metabolism, liver, serum, right brain, cerbellum, heart and adipose, and acquired lipidomics data on all. This included semi-quantitative data on around 1100 lipid isoforms, i.e. the species of each lipid class such as PC(34:1) or TG(48:0). This provided data on the expected destinations of fatty-acid-containing compounds, *viz*. structure (brain), fatty acid oxidation (heart) and storage (adipose), Fig. 1B. These data were analysed using Shared and Unique Structures Plots for tissue-tissue comparisons, and Lipid Traffic Analysis (Furse et al., 2021d, 2021b), with updated code (v2.3), for network analyses.

The network analysis tool, Lipid Traffic Analysis (LTA) characterised the number, type and abundance of lipids in and between tissues, and calculated the relationship between the lipids for the two groups (*e.g.* control and experimental). This allows us to use the spatial or temporal distribution of metabolites to characterise how the control of metabolism differs in a given phenotype. This approach is useful because it has found changes to the timing of lipid metabolism (Furse et al., 2021d) and changes in the tissues in which lipids accumulate (Furse et al., 2021b). LTA is crucial for testing the central hypothesis of the present study because it is the only tool that allows the investigation of the control of metabolism using metabolite abundance.

## Materials and methods

### Study design

The mouse model used in the present study is a model of paternal nutritional programming described in detail (Morgan et al., 2020; Watkins & Sinclair, 2014; Watkins et al., 2017, 2018) (Fig. 1A). This model was designed to test for a difference in dietary intake without a difference in calorific intake. Both diets were considered in the healthy range for protein and carbohydrate intake and were not obesogenic. Sperm and seminal plasma from each group were separated and used to produce four groups of offspring (See **Generation of F1 offspring**). Tissues were collected from F1 offspring to describe the lipid metabolism network (Fig. 1B) and lipidomics data collected (detailed description in the **Lipidomics** section below). The spatial distribution of lipids in the Control and Experimental groups’ networks was used to identify differences in the control of lipid metabolism between the two systems and thus test the hypothesis of the study. This method of data analysis, called Lipid Traffic Analysis (LTA) has been used previously by us to answer questions about paternal nutritional programming (Furse et al., 2021b), the present study is the first analysis that identifies differences between the propagation of programming effects between sperm and seminal plasma.

### Animal model

Animals were maintained at Aston University’s biomedical research facility as described previously (Watkins et al., 2018) and is shown in Fig. 1A in the context of the present study. Briefly, entire and vasectomised 8 week old C57BL6 males were fed either control normal protein, normal carbohydrate diet (NP-NC, ‘N’ in the present study; **18% casein**, 21% sucrose, 42% corn starch, 10% corn oil; *n* = 16 entire and 8 vasectomised males) or isocaloric low protein, high carbohydrate diet (LP-HC, ‘H’ in the present study; **9% casein**, 24% sucrose, 49% corn starch, 10% corn oil; *n* = 16 entire and 8 vasectomised males) for a period of 8–12 weeks. Diets were manufactured commercially (Special Dietary Services Ltd; UK) and their composition described previously (Watkins et al., 2018). The structure of the feeding, breeding and tissue collection were as previously described (Furse et al., 2021b).

### Generation of F1 offspring

Virgin 8-week-old female C57BL/6 mice (*n* = 8 litters per treatment) were super-ovulated by intraperitoneal injections of pregnant mare serum gonadotrophin (1 IU) and human chorionic gonadotrophin (1 IU) 46–48 h later. Intact NP-NC and LP-HC fed males were culled by cervical dislocation after a minimum of 8 weeks on respective diets. Sperm were isolated from caudal epididymi of NP-NC and LP-HC sires as described (Morgan et al., 2020; Watkins et al., 2018) and allowed to capacitate in vitro (37 °C, 135 mM NaCl, 5 mM KCl, 1 mM MgSO_4_, 2 mM CaCl_2_, 30 mM HEPES; supplemented immediately before use with 10 mM lactic acid, 1 mM sodium pyruvate, 20 mg.mL^−1^ BSA, 25 mM NaHCO_3_). Females were artificially inseminated 12 h post human chorionic gonadotrophin injection with ~ 10^7^ sperm and subsequently housed over night with a vasectomized C57BL/6 male fed either NP-NC or LP-HC diet. Females were weighed regularly (every 4–5 days) for the detection of weight gain associated with a developing pregnancy. Four groups of offspring were generated, termed NN (NP-NC sperm and NP-NC seminal plasma), HH (LP-HC sperm and LP-HC seminal plasma), NH (NP-NC sperm and LP-HC seminal plasma) and HN (LP-HC sperm and NP-NC seminal plasma). The number of females inseminated, pregnancy rates, gestation lengths and litter parameters have been reported (Watkins et al., 2018). In the current study, we focused on tissues collected from F1 and F2 NN (NP-NC) and LL (LP-HC) groups as these provide a model for normal- and high carbohydrate intake in humans, and in order to reduce complicating factors.

### Materials, animals, consumables and chemicals

Purified lipids were purchased from Avanti Polar lipids Inc. (Alabaster, Alabama, US). Solvents and fine chemicals were purchased from SigmaAldrich (Gillingham, Dorset, UK) and not purified further. Mice were purchased from Harlan Laboratories Ltd (Alconbury, Cambridgeshire, UK). Hormones were purchased from Intervet (Milton Keynes, UK).

### Lipidomics

Lipidomics data for this study were collected in the same analytical run as a previous study, using a combination of mass spectrometry and phosphorus NMR (Furse et al., 2021b). All procedures used are therefore precisely as already described (Furse et al., 2021b). Briefly, whole tissue/organ samples were homogenised in a chaeotropic buffer to prepare a stable, pipettable solution that was then extracted with a mixture of dichloromethane, methanol and triethylammonium chloride, with adjustments for the abundance of triglyceride in adipose (Furse et al., 2020a). Mass spectrometry samples were prepared and data collected in a high throughput fashion using Direct Infusion Mass Spectrometry (Harshfield et al., 2019), *via* glass-coated 384 well plates. NMR samples were prepared and data collected in a low throughput fashion using a modified (Furse et al., 2020a) form of the CUBO solvent system (Bosco et al., 1997; Cremonini et al., 2004; Culeddu et al., 1998; Murgia et al., 2003) and assigned using reference 2D spectra acquired for the purpose (Furse et al., 2020a, 2021c). Lipidomics data were interpreted using dual spectroscopy (Furse et al., 2020a), in the case of these data, a combination of DIMS and ^31^P NMR was used to establish the composition of phospholipids. Glycerides and cholesterol were identified and quantified based on the MS data only (Furse et al., 2021b). Lipid identification: 586 lipid variables in positive ionisation mode and up to 564 lipid variables in negative ionisation mode in liver, brain, heart and adipose homogenates and in serum were putatively identified according to the Metabolomics Standards Initiative at Level 2.

### Lipid traffic analysis

Lipid Traffic Analysis code v1.0 (Furse et al., 2021b) was further developed in the present study to produce Lipid Traffic Analysis code v2.3. Lipid variables in each compartment (lipid station) were categorised according to whether they are unique to it (***U ***type lipids), shared with one adjacent to it (***B ***type lipids, uni- and bidirectional) or found in all compartments (***A ***type lipids). The code for the Binary Traffic analysis (Switch Analysis) (Furse et al., 2021b) was updated to include alignment of lists and automated calculation of JTCs and *p* values from binary lists and improved categorisation of lipid variables (including assessment of all TG-derived glycerides). The configuration of the ***U***-lipid, ***A***-lipid and ***B***-lipid sections of the code was altered to make running any of the three individual parts of the code feasible. Novel code was written in R(v3.6.x) and processed in RStudio(v1.2.5x). The full code for Lipid Traffic Analysis v2.3 can be found in the *Supplementary Information*. Dimensions for the Abundance Analysis were calculated using Eqs. 1 and 2 from previous work (Furse et al., 2021b):

1 $$Margin \,change={\overline{x} }_{\text{E}}-{\overline{x} }_{\text{C}}$$

2 $$Error \, normalised \, fold \, change=\frac{{\mathrm{log}}_{10}\left(\frac{{\overline{x} }_{\text{E}}}{{\overline{x} }_{\text{C}}}\right)}{\left(\sqrt{\frac{\left({a}^{2} + {b}^{2}\right)}{2}}\right)}$$

The analysis of the present study used only MS data and was similar to previous studies (Furse et al., 2021b, 2022) but with adjustments for four rather than two groups. The tissues used were mapped to the known biological/metabolic network (Fig. 1B). Categories for the Switch Analysis were ***A***, ***B*** and ***U*** lipids. Dimensions for the Abundance Analysis were calculated using Eqs. 1 and 2 in the original LTA paper (Furse et al., 2021b). Variables were regarded as present if they had a signal strength > 0 in ≥ 66% of samples.

**Statistical methods.** Univariate and bivariate statistical calculations, and error normalised fold change (ENFC) (Furse et al., 2021b), were done in Microsoft Excel 2016. Graphs were prepared in OriginLab 2018. Calculations of Jaccard-Tanimoto Coefficients (JTCs) (Furse et al., 2021b; Jaccard, 1912; Tanimoto, 1958) and associated *p* values (Rahman et al., 2014) were used as a non-parametric measure of the distinctions between lipid variables associated with phenotype(s). The *p* value associated with each *J* represents the probability that the difference between the lists of variables for the two phenotypes occurred by random chance, representing both the number of variables only found in either of the two groups and the order of the binary list. They are not the same as the *p* values used in a student’s *t-*test. To guide interpretation, in an LTA, typically, a *p* value ≤ 0.55 means that at least one of the variables of each group does not appear in the other whereas a *p* value is 0, none of the variables in the two groups is the same.

## Results

**Fig. 2:**
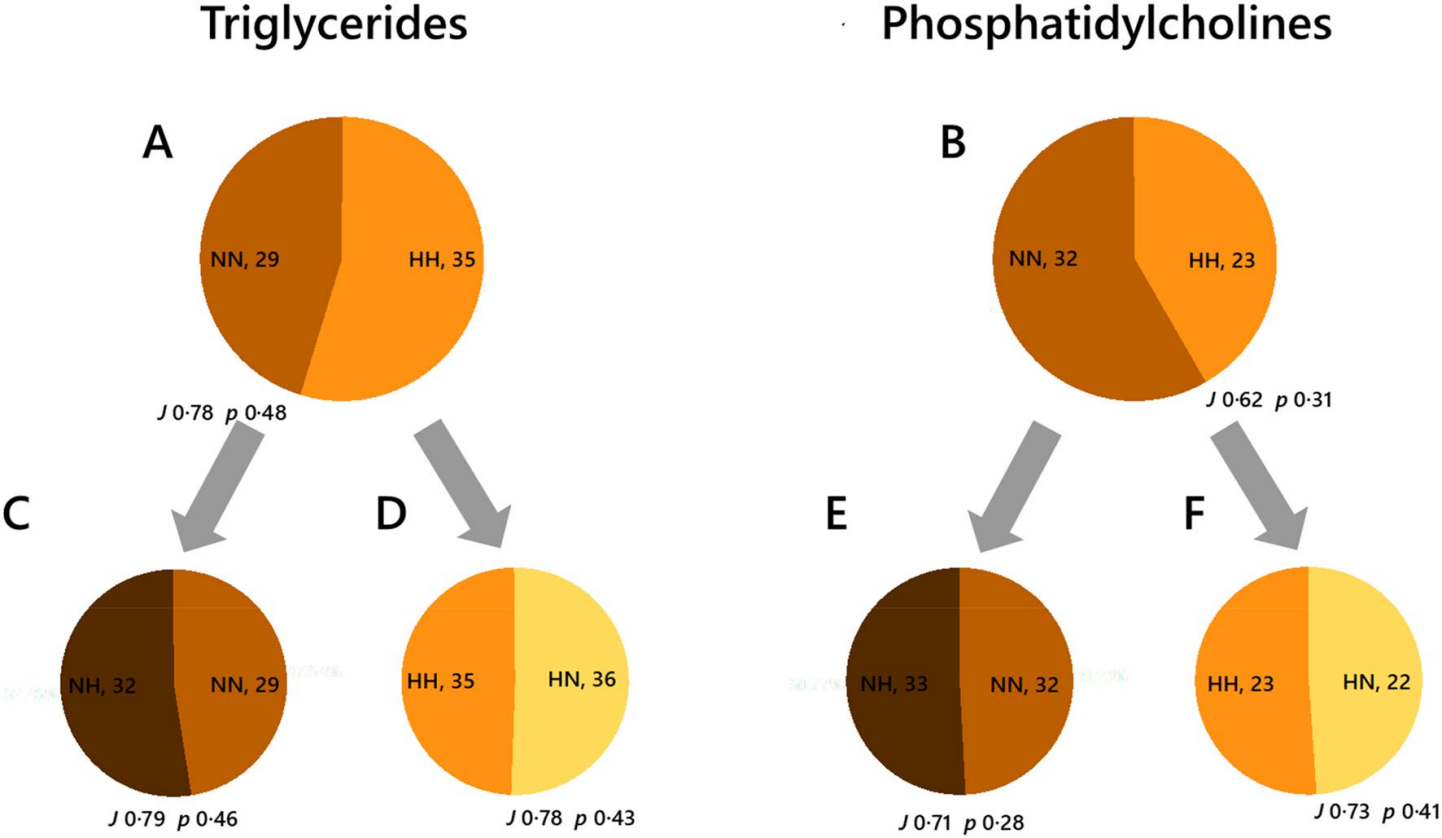
The ubiquitous triglyceride (TG) and phosphatidylcholine (PC) variables found in mice associated with a paternal high carbohydrate diet. Panel A, Triglycerides (NN and HH groups); B, Triglycerides (HN and HH groups); C, Triglycerides (NH and NN groups); D, Phosphatidylcholines (NN and HH groups); B, Phosphatidylcholines (HN and HH groups); C, Phosphatidylcholines (NH and NN groups). NN, NH, HN and HH refer to the feeding group of the (grand)sire from which the spermatozoa and seminal plasma were drawn, respectively. N, normal; H, high carbohydrate, low protein. The grey arrows show the comparisons of the control groups with the cross-paired sperm-seminal plasma. The Jaccard-Tanimoto coefficients (*J*) and probability (*p*) values that describe the similarity between sets of variables. Accompanying tables show the *J* and *p* values for sets of variables with seminal plasma from the same phenotype as the control where the number of variables is similar between the two groups

The mouse model showed differences in the number of A-type triglycerides (TG), i.e. TGs found throughout the biological networks (Fig. 1B) of the control feeding groups (NN and HH, Fig. 2A). The two diets, normal and high carbohydrate, led to different configurations of lipid metabolism in offspring. This was observed through the number of different isoforms of TG that appear throughout the network for NN and HH. The Jaccard-Tanimoto distance (*J*) and probability (*p*) statistics for the NN-HH comparison for the TGs in the two groups (*J* 0·78 and *p 0*·48, Fig. 2) showed that a majority of the 29 TG species found throughout the NN system were also found throughout the HH system. This was interpreted through the *J* and *p* statistics calculated; *J* represents the similarity factor of the two groups and *p* values represent the probability that the difference between the lists of variables for the two phenotypes occurred by random chance. The *p* values quoted are not the same as those of a student’s *t*-test and should not be interpreted as thresholds, though some general patterns exist. Where *p* ≤ 0·55, typically at least one variable in each group is not also in the other, and where *p* = 0, no variables are shared between the two groups. Thus, the *J* of 0·78 and *p* of 0·48 shown in Fig. 2 showed that there were 6 additional species in the HH group (29:35, the number of variables for the control and experimental groups, respectively) and that both systems have isoforms of TG that appear throughout it that the other group does not. These results about TG metabolism showed that the differences in dietary intake by fathers caused nutritional programming of lipid metabolism and differences in the distribution of energy storage lipids.

The role of phosphatidylcholines (PCs) was tested in the same way, Fig. 2B. The comparison of the paternal diet control groups (NN and HH) for PCs showed that there were fewer isoforms of PC that appeared throughout HH than NN (32:23) and several isoforms that only appeared in one of the two groups (*J* 0·62, *p* 0·31). PCs showed a similar trend to the TGs, with clear differences between the two dietary intakes. We therefore tested the primary hypothesis through a comparison of the two cross-paired feeding type phenotypes, with the controls (NN against NH, and HH against HN). These comparisons are designed to demonstrate whether the seminal plasma of the sire on a high carbohydrate diet modulated the profile of TGs in the offspring. This tests the primary hypothesis of the study as if the programming effects are propagated only through sperm, the source of the seminal plasma will not influence the lipid metabolism of the offspring.

The NH group showed a different profile of TG species to the NN group, as did the HN group from the HH group (Fig. 2C, D). The *J* and *p* statistics showed that there was an overlap of 0.79 between the NN and NH groups, with a *p* of 0·46 (Fig. 2C). This same pattern is observed in the HH against HN comparison and also for both comparisons for PCs (Fig. 2E, F). This result showed that the seminal plasma from normal and high carbohydrate-fed fathers has a different effect on the number of ubiquitous TGs and also that the two propagation routes alter it in different ways, which we had not expected.

**Fig. 3:**
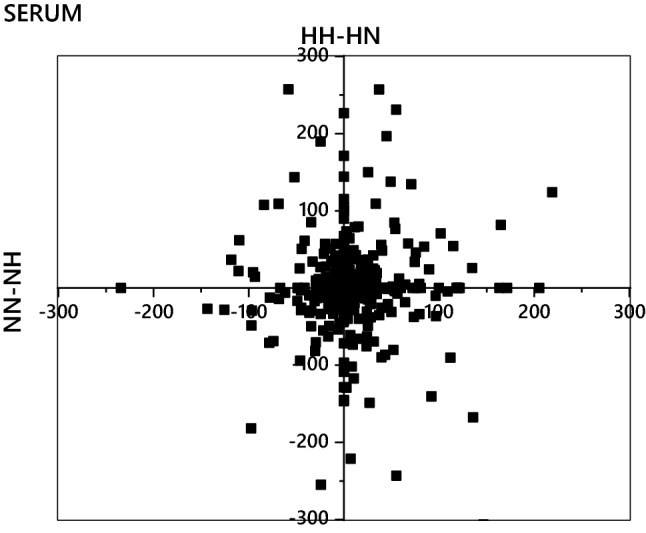
Shared and Unique Structures Plot of the error-normalised fold change for each variable measured in the serum of adult mice whose fathers were fed a high carbohydrate (H) or normal (N) diet. The two-letter codes represent the pairing of sperm and seminal plasma, *i.e*. NH represents sperm from sires fed a normal diet and seminal plasma from sires fed a high carbohydrate diet. ENFC calculated from two groups with the same sperm phenotype in order to test the hypothesis that the nutritional programming is propagated through sperm only. ENFC calculated from relative abundance of the variables (‰), calculated as previously described (Furse et al., 2021b)

The evidence for distinct propagation of nutritional programming effects through spermatozoa and seminal plasma in both TG and PC metabolism raised questions about how this affected individual tissues associated with lipid metabolism (and metabolic disease). We tested for tissue-tissue effects by constructing Shared and Unique Structures Plots (SUSPs; Fig. 3, *Fig. S1*). These SUSPs were used to identify lipid biosynthesis pathways, if any, that were affected by the paternal diet in individual tissues. Error normalised fold change (Furse et al., 2021b) (ENFC) was calculated for all variables and all compartments for NN-NH and HH-HN comparisons. The plot for serum (Fig. 3) showed the general pattern of these, with no clear evidence for a separate group of lipids associated with the effect (*Fig. S1*). The difference in the profile of both ubiquitous TGs and PCs between NN and NH, and HH and HN, together with the evidence that there were no clear effects within individual tissues, raised the question of how the effects of the parental diet can be characterised. We formed the hypothesis that there were changes to the metabolic machinery (biochemical infrastructure) of the system that exist throughout the system that are not measurable at a local level (through tissue-tissue comparisons).

**Fig. 4:**
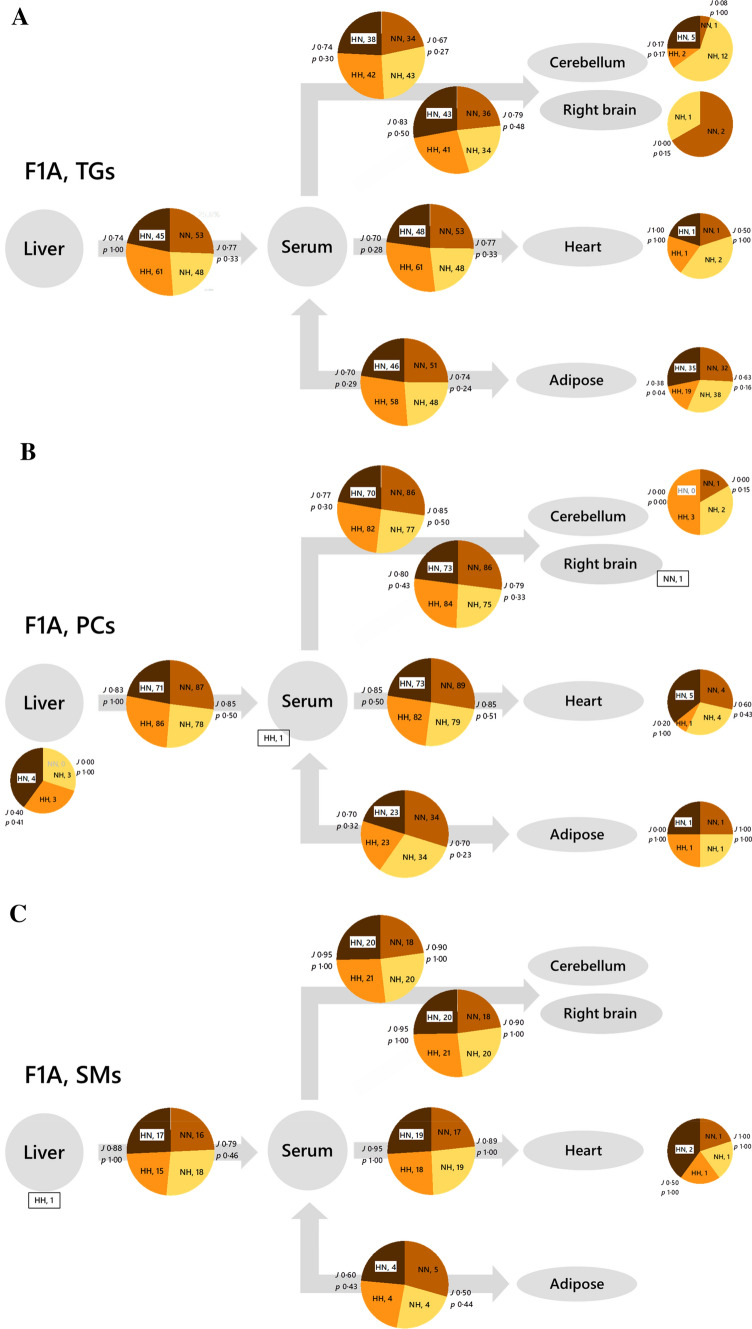
Switch analysis of B-type triglyceride (TG), phosphatidylcholine (PC) and sphingomyelin (SM) isoforms for F1 Adults. Panel **A**, Triglycerides (TGs). Panel **B**, Phosphatidylcholines (PCs). Panel **C**, Sphingomyelins (SMs). The Jaccard-Tanimoto coefficients (*J*) and probability (*p*) values that describe the similarity between sets of variables. Inset tables show the *J* and *p* values for sets of variables with seminal plasma from the same phenotype as the control where the number of variables is similar between the two groups. TGs were detected in positive ionisation mode whereas PCs were detected in negative ionisation mode

We used a Switch Analysis from LTA (v2.3, see *Methods*) to test this. We identified differences in the spatial distribution of lipids in the system. Switch Analysis showed that both TGs and PCs only found in part of the system followed a similar trend to the ubiquitous lipids of the same classes (shown in Fig. 2). There was typically an overlap of 0·7–0·8 for TGs and 0·8–0·9 for PCs, and with both possessing variables only found in one of the two phenotypes in each comparison, i.e. *p* < 0·55 (Fig. 4). A Switch Analysis of a third lipid class, sphingomyelins (SMs), showed that the differences in distribution of this lipid class are less strongly affected by the paternal diet, at least in parts of the system (Fig. 4C).

**Fig. 5:**
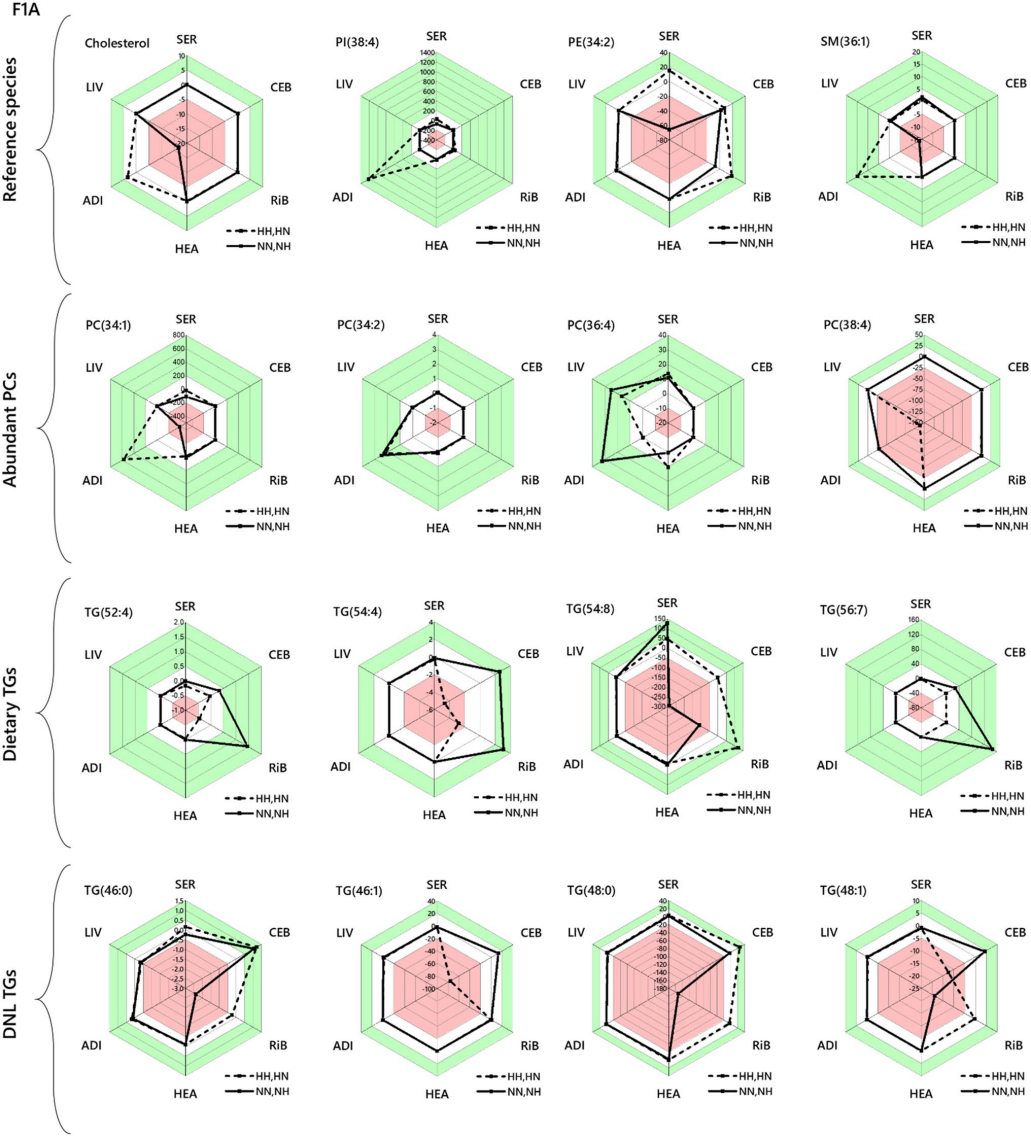
Abundance analyses of lipid variables in the neonate and adult F1 offspring of fathers fed a normal or a high carbohydrate diet, measured by mass spectrometry in positive ionisation mode. These radar plots show fold change in abundance of lipid variables unrelated (rows 1 and 2) and related (row 3) to *de novo* lipogenesis between two groups, scaled to the error. The value given is the log of the mean of experimental abundance values divided by the mean of control values, divided by the propagated error for that variable(Furse et al., 2021b). The values for the NN and HH groups were the denominators. NN, NH, HN and HH refer to the feeding group of the (grand)sire of the spermatozoa and seminal plasma, respectively. N, normal; H, high carbohydrate, low protein. ADI, adipose; CEB, cerebellum; HEA, heart; LIV, liver; RiB, right brain; SER, serum

The evidence for differences in the control of lipid metabolism in the phenotypes suggested to us that distribution rather than the activity of biosynthetic pathways may differ between phenotypes. We used an Abundance Analysis (Furse et al., 2021b) to quantify the difference in abundance of established molecular biomarkers across the network. This is a targeted analysis that augments the Switch Analysis by quantifying the abundance of lipid species with a known biological role or origin. The Abundance Analysis was done by calculating the ENFC (Furse et al., 2021b) of a set of variables representing major phospholipid classes and cholesterol; a set of variables associated with *de novo* lipogenesis (DNL), TG(46:0, 46:1, 48:0, 48:1) (Sanders et al., 2018); a set representing TGs associated with dietary intake, TG(52:2, 54:4, 54:8, 56:7) and the most abundant PCs, PC(34:1, 34:2, 36:4, 38:4). Radar plots of the analyses are shown in Fig. 5. The control groups (NN and HH) groups were tested against the groups that had spermatozoa from the same source but different seminal plasma (NH and HN, respectively). As with the tissue-tissue comparisons (SUSPs) and Switch Analyses, changes to lipid metabolism driven only by the spermatozoa would give a difference in ENFC for either of the two comparisons, but not both.

The Abundance Analysis showed that there is a clear difference in the effects on the distribution of structural lipids (such as PC) and energy storage and distribution (TGs) and where this occurs in the system. The adipose in particular shows a difference between comparisons for structural lipids and cholesterol (Fig. 5). However, there is not a clear pattern, even within PCs alone. This suggests that the composition of membranes in adipose is shaped by nutritional programming through seminal plasma. By contrast, adipose, liver and serum were largely similar in TG profile. The CNS tissues tested showed considerable differences in abundance of both DNL and dietary TGs between comparisons, showing here too that seminal plasma influences TG distribution within the CNS. Like PCs, there is also no clear pattern. The absence of a clear pattern for the abundance of PCs in adipose and TGs in the CNS explains why tissue-tissue comparisons failed to identify such differences. These results are also consistent with those above, that both seminal plasma and sperm propagate changes in lipid metabolism in offspring in a mouse model of high paternal carbohydrate intake.

## Discussion

Our study was motivated by the hypothesis that changes in lipid metabolism driven by the dietary intake of grandsires was propagated through spermatozoa rather than seminal plasma. This hypothesis was based on spermatozoa because the complicated changes to lipid metabolism driven by paternal nutrition might be expected only to be possible through subtle changes to gene expression. However, network analysis showed that the feeding group associated with both spermatozoa and seminal plasma shaped lipid metabolism in offspring, with effect sizes of a roughly similar breadth and magnitude but a different sort.

The evidence collected suggests that there are several effects on lipid metabolism propagated through both routes of spermatozoon and seminal plasma. The existence of an influence of each suggests that seminal plasma has a more important role in shaping lipid metabolism than has hitherto been realised. The result is robust partly because of the structure of the mouse model used in the present study. The sperm and seminal plasma were separated and combined as appropriate for each of the four groups and inseminated in the same manner for each. The specific effects on both structural lipids and energy storage and distribution invite speculation about the relationship between these and the changes to internal structures and volume of adipose (Arner et al., 2010; Gimpfl et al., 2017) in offspring associated with increased risk of metabolic disease such as type II diabetes mellitus. It is not currently possible to pinpoint that relationship precisely, however our results can inform the generation of hypotheses about how the membrane composition of adipocytes and in the CNS modulates their function in vivo and the role this may have in metabolic disease.

The propagation of nutritional programming effects through both the spermatozoon that fertilises the ovum and the seminal plasma delivered at the same time are undoubtedly of interest because the reach of the two is distinct. The spermatozoon has a fundamental impact on the genome of the resulting individual and thus its reach is entire. The seminal plasma has reach through its influence on the uterine environment. Previous reports have shown some of the downstream effects of the seminal plasma. Evidence from studies in mammals show that repeated exposure to seminal plasma leads to better fertilisation(Robertson & Sharkey, 2016) and a lower prevalence of inflammation in gestation (Bromfield, 2016; Kenny & Kell, 2018). Studies in *Bos taurus* have shown that the seminal plasma used can influence genes involved in the cell cycle and growth (Mateo-Otero et al., 2020). This evidence, along with that from the present study, suggests that seminal plasma shapes lipid metabolism by modulating the uterine environment during pregnancy. The reasons for the specificity to the developing individuals’ adipose and CNS are not clear. One possible explanation is that this effect is marked in these two compartments due to their heavy involvement in lipid metabolism, rather than a function-specific effect.

Importantly, our study shows that the effects of both routes include programming affects on biosynthesis as well as distribution, albeit with an unclear pattern in the former. It remains difficult to disentangle these, which is inherent in lipid metabolism as it is made up of several processes that are not linked directly. The evidence for changes to lipid biosynthesis came from Switch Analyses (Figs. 2 and 4) and Abundance Analyses (Fig. 5), without clear evidence of this within individual tissues (Fig. 3).

Switch Analyses showed that coverage between groups (the number of lipid variables found in both groups in a given comparison, measured by the Jaccard-Tanimoto coefficient or *J* value) was typically below 90% for both triglycerides and phosphatidylcholines, with different variables unique to either group (typically *p* values were below 0·55). The patterns observed in individual marker species were inconsistent with one another but differed considerably from no effect (ENFC = 0). This therefore shows that there are considerable changes to the lipid composition that exist within biosynthetic pathways, presumably affecting it at several points, *e.g.* the availability of fatty acids and the interconversion of lipid head groups. As the abundance of cholesterol is also programmed with this phenotype, sterol synthesis or possibly the enteric circulation may be altered too. Further work with a different model is required to separate the possible changes to lipid metabolism wrought through sperm and seminal plasma, *e.g.* fatty acid effects and head group biosynthesis/interconversion.

## Conclusions

This study shows that the hypothesis that lipid metabolism is altered by sperm alone was not correct. Instead, the novel LTA v2.3 showed that both possible channels for propagation, sperm cells and seminal plasma, are involved in shaping the control over phospholipid, triglyceride an even cholesterol metabolism in offspring. It also suggests that the compartments that are the most heavily involved in lipid metabolism (adipose and CNS rather than heart) are the ones in which the effect was strongest. However, that effect was counter-intuitive as it was the structure of the adipocytes and the abundance of TGs in the CNS that was affected, rather than the other way around. This study paves the way for investigations of the relationship- between paternal programming and the risk of CMD in offspring. Such studies would be important because they could inform interventions to overcome an increased risk of programmed metabolic disease that have been driven by parental diet.

## Supplementary Information

Below is the link to the electronic supplementary material.

Supplementary file1 (DOCX 86 KB)

Supplementary file2 (TXT 29 KB)

Supplementary file3 (XLSX 124 KB)

Supplementary file4 (PDF 368 KB)

## Data Availability

The supplementary figures are in *Supplementary file 1*. The novel R code developed in the present study for Lipid Traffic Analysis v2.3 is in *Supplementary file 2*. LTA v1.0 is available publicly available through Zenodo (Furse et al., 2021a) and BioRxiv (Furse et al., 2020b). The abundance analysis for the present study is shown in *Supplementary file 3*. The MS dataset used was generated in a previous study (Furse et al., 2021b). The MS lipidomics dataset used in the present study is available from the corresponding author on reasonable request, with annotations of signals shown in *Supplementary file 4*. The original NMR data acquired for the samples used in the study is available publicly (Furse et al., 2021b). No unique reagents were generated in this study.

## Abbreviations

CMD: Cardio-metabolic disease
CNS: Central nervous system
DNL: *De novo* Lipogenesis
ENFC: Error-normalised fold change
HH: Offspring group bred from sperm from fathers fed a high carbohydrate, low protein diet and seminal plasma from ones fed a high carbohydrate, low protein diet
HN: Offspring group bred from sperm from fathers fed a high carbohydrate, low protein diet and seminal plasma from ones fed a normal diet
JTC: Jaccard-Tanimoto coefficient
LTA: Lipid Traffic Analysis
NH: Offspring group bred from sperm from fathers fed a normal diet and seminal plasma from ones fed a high carbohydrate, low protein diet
NN: Offspring group bred from sperm from fathers fed a normal diet and seminal plasma from ones fed a normal diet
MS: Mass Spectrometry
PC: Phosphatidylcholine
PE: Phosphatidylethanolamine
PI: Phosphatidylinositol
SM: Sphingomyelin
SUSP: Shared and unique structures plot
TG: Triglyceride

## Declarations

### Research involving human/animal participants

Animals were maintained at Aston University’s biomedical research facility.
