# Supplementary material for: Paternal nutritional programming of lipid metabolism is propagated through sperm and seminal plasma
Source: Metabolomics. 2022 Feb 10;18(2):13. doi: 10.1007/s11306-022-01869-9 (PMC8828597; doi:10.1007/s11306-022-01869-9)
Supplement: Supplementary file 1 — Supplementary file1 (DOCX 86 KB) [file 11306_2022_1869_MOESM1_ESM.docx]

**Supplementary Figures and Tables**


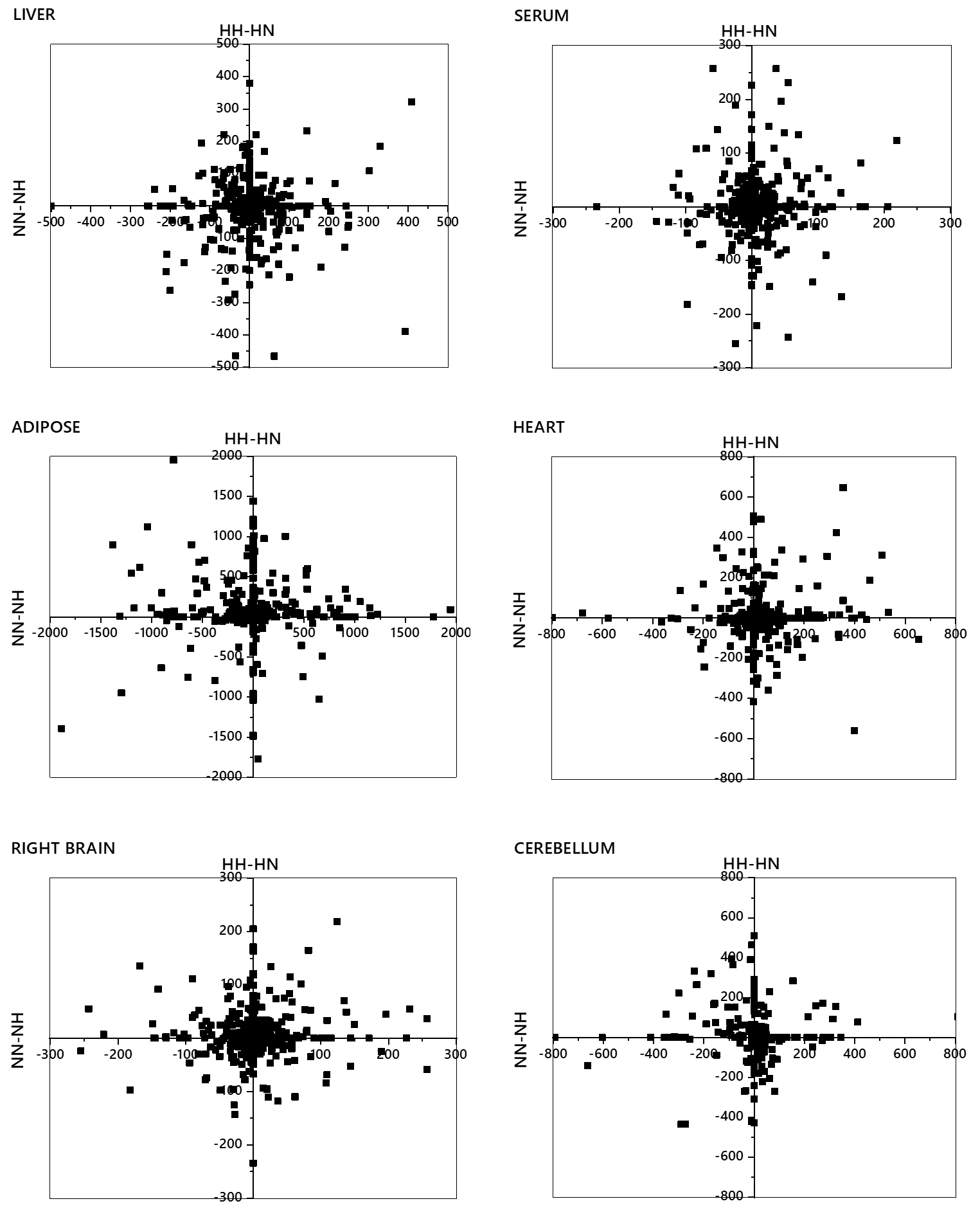


**Fig. S1. Shared and Unique Structures Plots of the Error Normalized Fold Change for comparisons of lipid abundance in control (NN or HH) groups with cross-paired groups (NH and HN).** Each dot shows one lipid variable. The absence of clear subgrouping or a coherent pattern in any tissue led to the conclusion that tissue-tissue effects were not significant. N, normal diet; H, high carbohydrate, low protein diet (fed to the fathers of the individuals profiled). Two letter codes indicate the source of the sperm (first letter) and the seminal plasma (second latter) respectively.

**Supplementary Materials**

1. Supplementary Data file S1. Lipid traffic Analysis v2.3 (R code for conducting Lipid Traffic analysis.)
2. Supplementary Data file S2. Abundance analysis (ENFC).
3. Supplementary Data file S3. Annotations of signals.

References

Furse, S., et al. (2021). Lipid Traffic Analysis reveals the impact of high paternal carbohydrate intake on offsprings’ lipid metabolism. Communications Biology, doi:10.1038/s42003-021-01686-1
